# Sirt1 enhances tau exon 10 inclusion and improves spatial memory of Htau mice

**DOI:** 10.18632/aging.101564

**Published:** 2018-09-22

**Authors:** Shuo Qian, Jianlan Gu, Wufei Dai, Nana Jin, Dandan Chu, Qin Huang, Fei Liu, Wei Qian

**Affiliations:** 1 Department of Biochemistry and Molecular Biology, Medical School, Nantong University, Nantong, Jiangsu 226001, P. R. China; 2 Jiangsu Key Laboratory of Neuroregeneration of Jiangsu and Ministry of Education of China, Co-innovation Center of Neuroregeneration, Nantong University, Nantong, Jiangsu 226001, P. R. China; 3 Department of Neurochemistry, Inge Grundke-Iqbal Research Floor, New York State Institute for Basic Research in Developmental Disabilities, Staten Island, NY 10314, USA; * Equal contribution

**Keywords:** Sirt1, resveratrol, 9G8, tau exon 10 splicing, Alzheimer’s disease

## Abstract

Alternative splicing of tau exon 10 generates tau isoforms with three or four microtubule binding repeats, named 3R-tau and 4R-tau, respectively. Dysregulation of tau exon 10 splicing could cause neurofibrillary degeneration. Acetylation is one of the major post-translational protein modifications in the cell by attachment of the acetyl group to either the α-amino group of the N-terminus of proteins or to the ε-amino group of lysine residues. Sirt1, one member in mammalian Sirtuin family, deacetylates protein and is associated closely with age-related diseases including Alzheimer’s disease. However, the role of Sirt1 in tau exon 10 splicing remains elusive. In the present study, we determined the role of Sirt1 in tau exon 10 splicing. We found that activation of Sirt1 by resveratrol enhanced tau exon 10 inclusion, leading to 4R-tau expression. Sirt1 interacted with splicing factor 9G8, deacetylated it at Lys24, and suppressed its function in promoting tau exon 10 exclusion. Moreover, resveratrol improved learning and spatial memory in Htau mice. These findings suggest that Sirt1 may serve as a new drug target for Alzheimer’s Disease related tauopathies and resveratrol may be used to correct dysregulated tau exon 10 with 3R-tau > 4R-tau.

## Introduction

Microtubule-associated protein tau promotes tubulin assembly and stabilizes microtubules structure. Tau gene, *MAPT*, is located on the long arm of human chromosome 17q21 [1]. There are six isoforms of tau proteins expressed in normal human brain by the alternative splicing of exons 2, 3, and/or 10 of tau pre-mRNA [2]. Exon 10 encodes the second microtubule-binding repeat. Its alternative splicing generates tau isoforms with three or four microtubule binding repeats, called 3R- or 4R-tau [3]. In adult human brain, the ratio of 3R to 4R is about 1:1 [2]. Almost half of tau gene mutations associated with FTDP-17T (frontotemporal dementia with Parkinsonism linked to chromosome 17 and specifically characterized by tau pathology) only disturb exon 10 splicing to disrupt the balance of 3R to 4R tau [4]. In addition to FTDP-17T, the imbalance of 3R-tau and 4R-tau expressions has been reported in several other tauopathies [5,6]. Thus, abnormal exon 10 splicing is sufficient to cause neurodegeneration and dementia [5]. Maintaining the balance of 3R-tau and 4R-tau is important for normal brain function.

Sirtuins (Sirt1-Sirt7) are class III histone deacetylases (HDACs) requiring NAD^+^ for their activity [7]. Targets of mammalian Sirt1, one of Sirtuins, are important in energy metabolism, circadian rhythm, and aging [8,9]. Sirt1 alleviates AD pathologies via reducing amyloid plaques and suppressing symptoms related to tau effectively [10]. Sirt1 activation may become the new strategy for preventing neurodegenerative disorders [11]. Resveratrol, a polyphenol found in red grapes, red wine, and other plant foods, is the most potent molecule for Sirt1 activation [12]. Resveratrol treatment decreases age-dependent cognitive decline and AD-like pathologies in animal models [13]. Resveratrol suppresses cognitive decline through promoting Sirt1 expression or the activation of Sirt1 [14].

Splicing factor 9G8 regulates both constitutive splicing and alternative splicing of many pre-mRNAs [15]. 9G8 can facilitate mRNA translocate to the cytoplasm and promote the expression of unspliced mRNA 9G8 may directly bind to the proximal downstream intron of tau exon 10 and inhibit tau exon 10 inclusion [16]. It is well known that the localization and functions of 9G8 are firmly regulated by phosphorylation [17]. Overexpression of dual-specificity tyrosine-phosphorylated and regulated kinase 1A (Dyrk1A) makes 9G8 translocate to cytosome and reduce its activity in tau splicing. Transcriptional factors and splicing factors are modified by acetyl group and acetylation affects their functions [18,19]. The role of Sirt1 in tau splicing remains elusive.

Acetylation introduces an acetyl functional group into a chemical compound whereas deacetylation is the removal of an acetyl group. Acetyltransferases catalyze the transfer of an acetyl group from a donor molecule, deacetylases catalyze the reversal of acetylation. The α-amino group of the first amino acid of protein is acetylated [20] and proteins are also acetylated on ε-amino group of lysine (K)residues [19,21]. Acetylation occurs as a co-translational and post-translational modification of proteins, acetylation has a considerable impact on gene expression and metabolism [22]. It has been reported that reduction of the tau acetylation may lead to effective treatments for cognitive decline in AD [23].

In current research, we determined the role of Sirt1 in the alternative splicing of tau exon 10. We found that Sirt1 enhanced tau exon 10 inclusion. Sirt1 deacetylated 9G8 and suppressed its function in facilitating tau exon 10 exclusion. Resveratrol treatment enhanced 4R-tau expression and improves spatial memory of Htau mice. These results suggest that Sirt1 enhances the inclusion of tau exon 10 via acting on 9G8, and serves as therapeutic target for tau exon 10 exclusion related tauopathies.

## RESULTS

### Resveratrol enhances Sirt1 expression and suppresses 3R-tau expression

**Figure 1:**
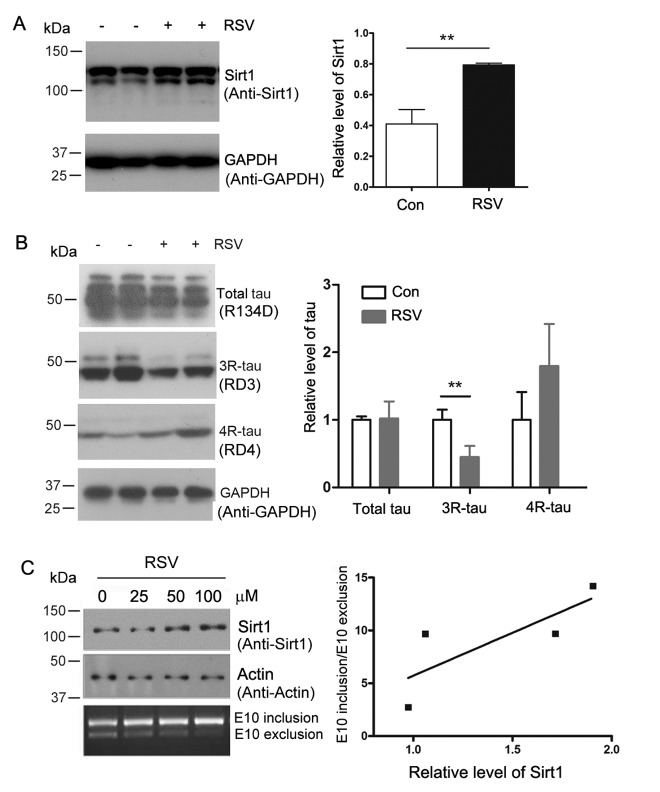
**Resveratrol enhances Sirt1 expression and suppresses 4R-tau expression.** (**A**, **B**) 5-month old Htau mice were treated with resveratrol for 7 months. The levels of 3R-tau, 4R-tau and total tau in the brains were analyzed by western blot developed with anti-Sirt1 (**A**) and anti-3R-tau and anti-4R-tau (**B**). The data are presented as mean ± S.D. (n=3-5) and analyzed by student *t*-test. **, p<0.01. (**C**) pCI/SI9-SI10 was transfected into HEK-293T cells and then the cells were treated with 0, 25, 50 and 100 μM of resveratrol for 48 h. Total RNA was extracted and alternative splicing of tau exon 10 was analyzed by RT-PCR. Ratio of exon 10 inclusion/exon 10 exclusion was plotted against the resveratrol concentration.

Htau mice are generated by crossing mice that express a human MAPTau H1 gene with tau knockout (KO) mice. The expression level of 3R-tau is significantly higher than that of the 4R-tau in the Htau mice [24]. In animal models, resveratrol treatment could decrease age-dependent cognitive decline [25]. To investigate whether resveratrol regulates the alternative splicing of tau exon 10, we fed 5-month old Htau mice and tau-KO mice with resveratrol at 20 mg/kg/day for 7 months. Since Sirt1 is the core effector of resveratrol [26], we analyzed the expression of Sirt1 in resveratrol treated and control mice by western blot developed with anti-Sirt1. We found that resveratrol enhanced the expression of Sirt1 in Htau transgenic mice brain (Fig. 1A).

Then the homogenates of mice brain were subjected to western blot analysis developed with anti-3R-tau antibody (RD3). We found that the protein expression level of 3R-tau was repressed by resveratrol obviously (Fig.1B). These data suggested that resveratrol inhibited the expression of 3R-tau in Htau mice brain.

We investigated the role of resveratrol on tau exon 10 splicing in cells. Mini-tau gene pCI/SI9-SI10, consisting of tau exons 9, 10 and 11, part of intron 9 (SI9) and intron 10 (SI10) was used to transfect into HEK-293FT cells and cells were treated with different concentration of resveratrol. After 48 h, total RNA was extracted and the alternative splicing products of tau exon 10 were examined by RT-PCR and Sirt1 was detected by western blot developed with anti-Sirt1 antibody. We found that the increased protein expression level of Sirt1 triggered by resveratrol could promote the inclusion of tau exon 10 (Fig. 1C). These data support that resveratrol increases expression of Sirt1 and promotes the inclusion of tau exon 10.

### Sirt1 suppresses 9G8-mediated tau exon 10 exclusion

**Figure 2:**
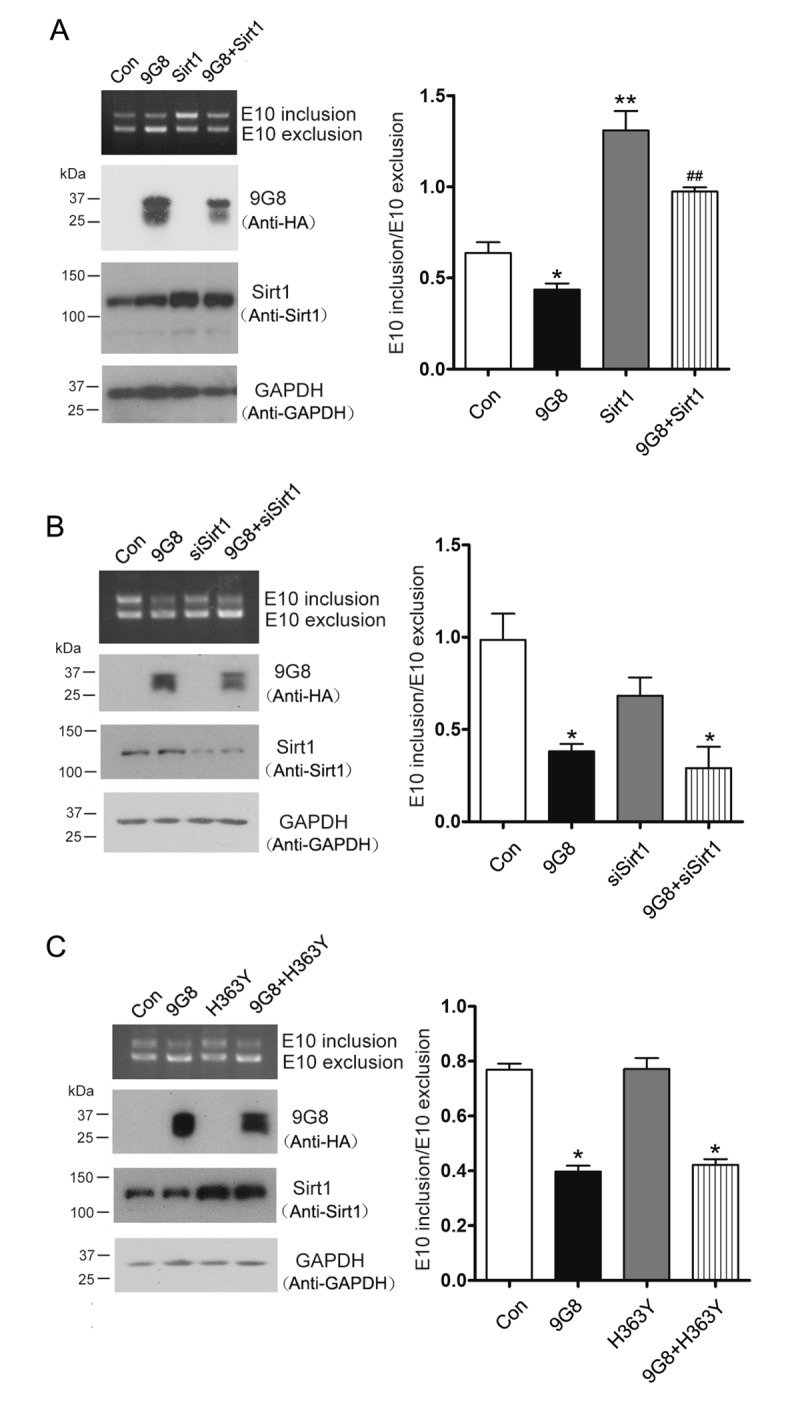
**Sirt1 inhibits 9G8-enhanced 3R-tau expression.** (**A**) 9G8 or Sirt1 was overexpressed individually or together into HEK-293T cells transfected with mini-tau gene pCI/SI9-SI10 for 48 h. The splicing products of tau exon 10 were analyzed by RT-PCR. (**B**) Mini-tau gene was co-transfected into HEK-293T cells with siRNA of Sirt1 only or together with 9G8, and then splicing products were analyzed by RT-PCR 48 h after transfection. (**C**) pcDNA3.1/SIRT1 or pcDNA/3.1H363Y was transfected only or together with 9G8 into HEK-293T cells. Splicing products were analyzed by RT-PCR after 48 h transfection. The ratios of tau exon 10 inclusion to exclusion are presented as mean ± S.D. and analyzed with two-way ANOVA. *, p < 0.05, **, p < 0.01, ***, p < 0.001, compared with control group; ^##^ p < 0.01, compared with 9G8 group.

9G8 promotes 3R-tau expression through inhibiting the inclusion of tau exon 10 [17,27]. 9G8 is reported to be acetylated at K24 [18], we want to know whether Sirt1, a deacetylase, is involved in the regulation of 9G8-mediated alternative splicing of tau exon 10. We overexpressed Sirt1 and 9G8 respectively or in combination in HEK-293T cells transfected with pCI/SI9-SI10. Splicing products of tau exon 10 were then measured by RT-PCR. We found that co-overexpression of Sirt1 with 9G8 prevented 9G8’s inhibition of tau exon 10 inclusion significantly (Fig.2A). We also knocked down the expression of Sirt1 by its siRNA and simultaneously overexpressed 9G8 in pCI/SI9-SI10 transfected HEK-293T cells. Then splicing products were quantified by RT-PCR. We observed that siSirt1 had no effect on the 9G8-promoted 3R-tau expression (Fig. 2B). To investigate whether Sirt1’s role in 9G8-mediated tau exon 10 splicing was dependent on its deacetylase activity, we co-transfected cells with 9G8 and the deacetylase-dead Sirt1 (H363Y). We found that co-transfection with H363Y did not affect the activity of 9G8 in tau exon 10 splicing (Fig. 2C). These results suggest that Sirt1 suppresses the role of 9G8 in promoting 3R-tau expression relying on its deacetylase activity.

### Sirt1 interacts with 9G8 and deacetylates 9G8

**Figure 3:**
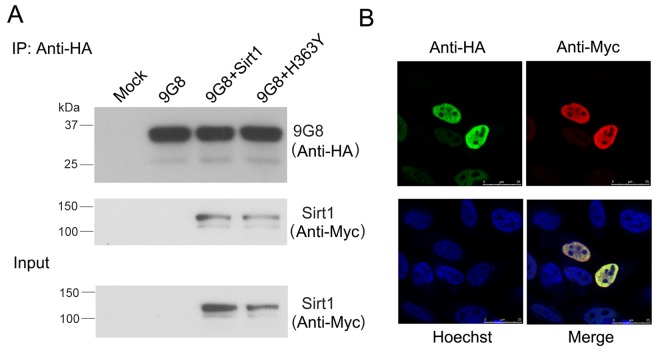
**Sirt1 interacts with 9G8 directly.** (**A**) Myc-tagged SIRT1 and HA-tagged 9G8 were co-expressed in HEK-293T cells. After 48 h transfection, cell lysate was incubated with anti-HA antibody coupled onto protein G beads. The proteins bound on beads were analyzed by western blot developed with anti-HA and anti-Myc. (**B**) Sirt1 tagged with Myc and 9G8 tagged with HA were co-overexpressed in Hela cells. After 48 h transfection, the cells were fixed and immunostained with anti-HA or anti-Myc and followed by TRITC-anti-rabbit IgG or FITC-anti-mouse IgG. Hoechst was used for nuclear staining.

We investigated whether 9G8 and Sirt1 interact with each other. We performed co-immunoprecipitation in cultured cells to validate the interaction between Sirt1 and 9G8. Sirt1 could be co-immunoprecipitated with HA-9G8 by anti-HA antibody (Fig. 3A). These results indicate the interaction between 9G8 and Sirt1. To study the interaction of 9G8 with Sirt1 in live cells, we co-expressed HA-9G8 and Sirt1 in HeLa cells and then immunostained the cells with anti-HA and anti-Myc antibody. Their subcellular localization was examined by confocal microscopy. Both Sirt1 and 9G8 were colocalized in the speckles in the nucleus. These results provide further evidence of their interaction in cultured cells (Fig. 3B).

**Figure 4:**
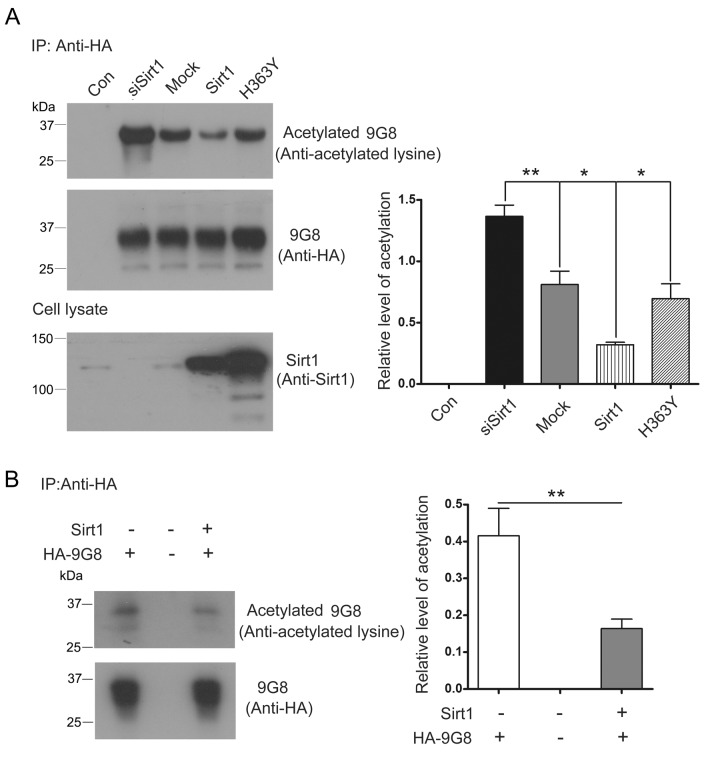
**Sirt1 deacetylates 9G8.** (**A**) HA-9G8 was co-transfected with pcDNA3.1/SIRT1 or pcDNA3.1/H363Y or siRNA of Sirt1 into HEK-293T cells. The cell extract was incubated with anti-HA precoupled to protein G beads. The bound proteins were subjected to western blots using anti-HA or anti-acetylated-lysine. The data are presented as mean ± S.D. (n=3) and analyzed with one-way ANOVA. **, p<0.01, *, p<0.05. (**B**) Immunoprecipitated HA-9G8 as described in A was incubated with or without Sirt1 in the presence of NAD^+^ for 2 h at 37°C. The level of 9G8 acetylation was analyzed by western blot developed with anti-HA and anti-acetylated-lysine and is presented as mean ± S.D. (n=3) and analyzed with student *t*-test. **, p<0.01.

The biological activity of 9G8 is inhibited by Sirt1. To investigate whether Sirt1 deacetylates 9G8, we co-transfected pCEP4/9G8-HA with pcDNA3.1/SIRT1, pcDNA3.1/H363Y or siSirt1, and then immunoprecipitated 9G8 with anti-HA antibody. The level of acetylated 9G8 was analyzed by western blot developed with anti-acetylated-lysine antibody. We found that the level of acetylated 9G8 was obviously decreased in the cells with overexpression of Sirt1 and significantly increased in the cells with knockdown of Sirt1 by siRNA. Moreover, overexpression of H363Y, deacetylase-dead mutant of Sirt1, had no effect on the acetylation status of 9G8 (Fig. 4A). These results indicate that Sirt1 deacetylates 9G8.

To study whether Sirt1 deacetylates 9G8 directly, deacetylation assay was performed in vitro. We incubated purified HA-9G8 with Sirt1 in vitro. Compared with no Sirt1 control, the acetylation level of 9G8 was decreased in the reaction mixture in the presence of Sirt1 (Fig. 4B). These results convince that HA-9G8 is deacetylated by Sirt1.

### Sirt1 deacetylates 9G8 at Lys24

**Figure 5:**
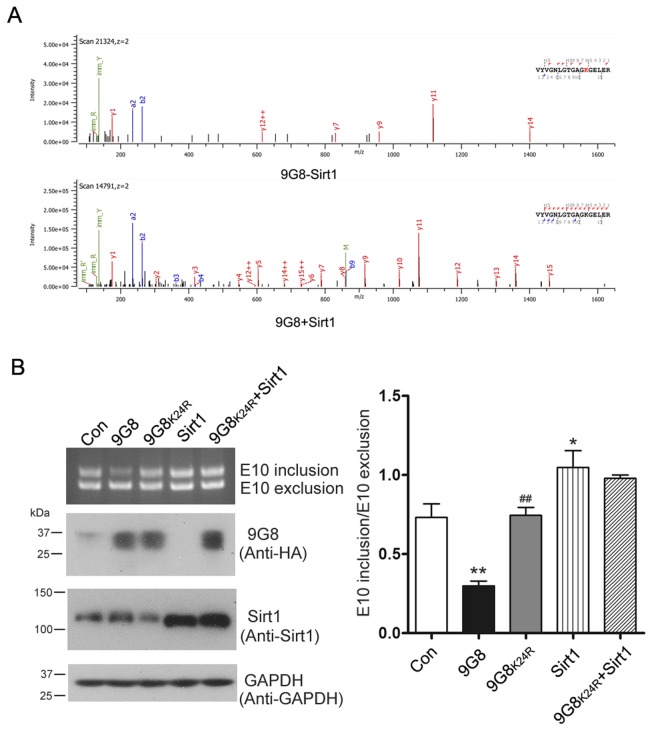
**Sirt1 deacetylates 9G8 at K24.** (**A**) Mass spectra obtained from HA-9G8 immunoprecipitated from HEK-293T cells co-transfected with pcDNA3.1/SIRT1 or control DNA. (**B**) Sirt1 was expressed alone or co-overexpressed with 9G8_K24R_ in HEK-293T cells transfected with pCI/SI9-SI10. After 48 h transfection, the alternative splicing products of tau exon 10 were analyzed by RT-PCR. The data are presented as mean ± S.D. (n=3) and analyzed with two-way ANOVA. * p < 0.05, ** p < 0.01, *** p < 0.001, compared with control group; ^##^ p < 0.01, compared with 9G8 group.

To identify 9G8's deacetylation sites by Sirt1, we immunoprecipitated HA-9G8 from HEK-293T cells overexpressing Sirt1 or not. The immunoprecipitated protein was resolved by SDS-PAGE, and then subjected to in-gel trypsin digestion and LC-MS/MS. The lysine residue in peptide fragment VYVGNLGTGAGKGELER was detected acetylated in 9G8 in absence of Sirt1 but not in 9G8 co-overexpressed with Sirt1 (Figure 5A). These results indicate that Sirt1 deacetylates 9G8 at K24.

To learn the role of 9G8 acetylation at K24, we mutated lysine 24 residue of 9G8 into arginine (R) as deacetylation mimetic, we co-transfected pCEP4/9G8 or pCEP4/9G8_K24R_ together with pCI/SI9-SI10 into HEK-293T cells. We analyzed the alternative splicing products of tau exon 10 by RT-PCR 48 h after transfection. We found that 9G8 but not 9G8_K24R_ inhibited the inclusion of tau exon 10 (Fig. 5B). These data suggest that acetylation of 9G8 at K24 was important for the function of 9G8 in tau exon 10 splicing. We also co-overexpressed 9G8_K24R_ and Sirt1 in HEK-293T cells transfected with pCI/SI9-SI10. We found that Sirt1 had almost no effect on the role of 9G8_K24R_ in regulating the splicing of tau exon 10 (Fig. 5B). These results further confirm that Sirt1 deacetylates 9G8 at K24 to suppress the 9G8-mediated splicing of tau exon 10.

### Resveratrol improves spatial memory of Htau mice

**Figure 6:**
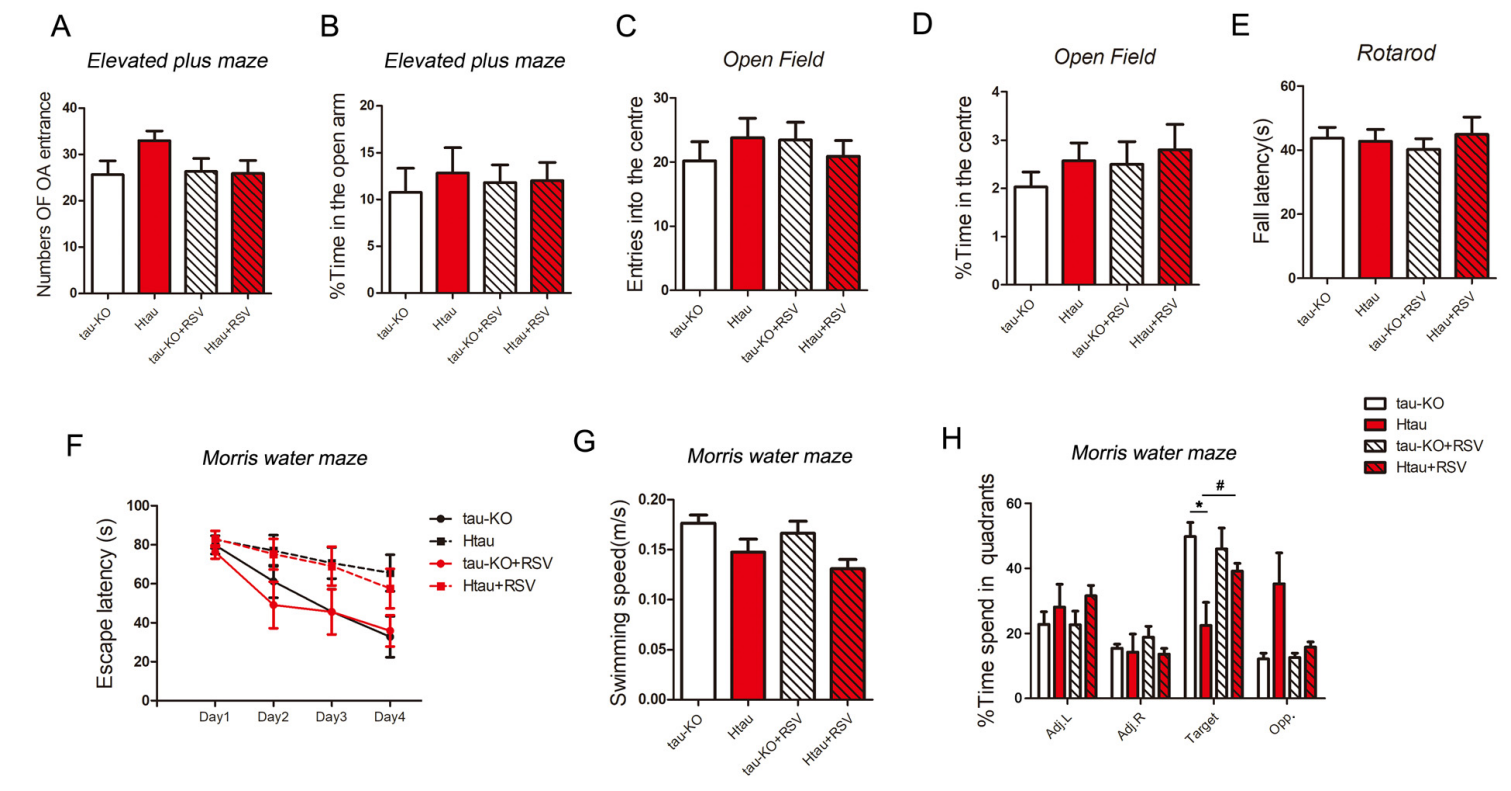
**Resveratrol rescues the impaired spatial memory of Htau mice.** (**A**-**E**) 5-month old tau-KO and Htau mice were treated with resveratrol for 7 months and subjected to general behavior tests. For elevated plus maze test, the amount of time spent in the open arm and number of open arm (OA) entrances were analyzed (**A** and **B**). For open field test, time amount spent in center area and entered into the center area were analyzed (**C** and **D**). Locomotor activity by tested rotarod test and fall latency were recorded (**E**). (**F**-**H**) Htau and tau-KO mice were subjected to Morris water maze test. During the training phase, travel latency (**F**) and swimming speed (**G**) were recorded and analyzed. In the probe trial, the time spent in each quadrant was recorded (**H**). The data are presented as mean ± S.D. (n=9-11) and analyzed by two-way ANOVA followed by Bonferroni post-test (n=9-11). *, p<0.05.

To investigate whether Htau mice, in which the expression of 3R-tau is more than that of 4R-tau, display abnormal general behavior, we studied the anxiety with the elevated plus-maze task and open-field task in Htau mice using tau-KO as control at 12-month old. We used accelerating rotarod test to determine the locomotivity and motor coordination. In all these general behavior test, Htau mice showed no significant difference compared with tau-KO mice (Fig. 6A-E).

Then, we evaluated the learning and memory of Htau mice by Morris Water Maze test at age of 12 months using tau-KO as control. Performance of animals during training was analyzed as latency to reach the submerged platform. On the premise of the same swimming speed (Fig. 6G), both Htau and tau-KO mice spent less time to find platform and traveled shorter distance at day 4 than that at day 1 (Fig. 6F), suggesting both of them had learning ability. But Htau mice spent more time (Fig. 6F) to reach the platform during training phase compared with tau-KO, displaying spatial learning defects in Htau mice.

We performed probe trials one day after the training trials to assess the spatial memory of the mice. On the probe trials, Htau mice spent less time in the target quadrant (Fig. 6H), suggesting the spatial memory is also impaired in Htau mice.

## DISCUSSION

Approximately equal levels of 3R-tau and 4R-tau are essential for normal brain function. Abnormal tau exon 10 splicing is sufﬁcient to cause neurodegeneration and dementia [28]. Imbalance of 3R-tau to 4R-tau also results in neurodegeneration in experimental animals. For example, the Htau transgenic mice, expressing the human *MAPT* gene in a null mice *Mapt* background, have more 3R-tau than 4R-tau [24]. Htau mice produce age-dependent tauopathy including tau hyperphosphorylation and aggregation as well as extensive neuronal death [24,29]. In the present study, we employ Htau mice as animal model to investigate whether and how Sirt1 corrects tau exon 10 mis-splicing and then improves the ability of spatial memory in Htau mice.

SR proteins are crucial in the regulation of tau exon 10 splicing. Different SR proteins modulate the alternative splicing differently [28]. For instance, SC35 promotes tau exon 10 inclusion, 9G8 induces its exclusion. Serine and threonine residues that can be phosphorylated are rich in SR proteins. The phosphorylation modification tightly regulates the localization and activity of SR proteins [28]. SC35 is also modified by acetylation [30,31]. Acetylation level regulates the role of SC35 in tau exon 10 splicing [31]. In this paper, we found that 9G8 is acetylated at K24 and its acetylation status has impact on tau exon 10 splicing.

The potential role of Sirt1 in AD and other neurodegenerative disorders has been addressed [32]. We found that Sirt1 deacetylated both SC35 and 9G8 to adjust the balance of 3R-tau/4R-tau. Previously, Sirt1 was shown to deacetylate SC35 to inhibit SC35-promoted 4R-tau expression [31]. Present study indicates for the first time that Sirt1 deacetylates 9G8 at K24 to suppress 9G8-enhanced 3R-tau expression.

Resveratrol works as the most potent activators for Sirt1 [13]. Resveratrol also activates AMPK [33,34]. Our data show that resveratrol increases the protein expression level of Sirt1 to reduce the ratio of 3R-tau to 4R-tau in Htau mice (Fig.1). The same effect of Sirt1 on the balance of 3R-tau/4R-tau is indicated in HEK -293FT cells (Fig.1). It has been indicated that resveratrol can cross the blood–brain barrier, resulting in detectable parent molecule in the brain [13]. In our study, resveratrol was administrated to 5-month old Htau mice in feeding water for 7 months. After 7 months treatment, elevated protein level of Sirt1, decreased 3R-tau were detected in brain extract of Htau mice (Fig.1). Resveratrol also improves spatial memory ability of Htau mice (Fig. 6). These data suggest that in Htau mice, ratio imbalance of 3R-tau to 4R-tau may lead to impairment of spatial memory and meanwhile resveratrol may improve spatial memory defects through balancing 3R-tau to 4R-tau.

In mass spectrometry analysis, 9G8 proteolytic peptides were from tryptic-digestion. The middle and C-terminal region of 9G8 was rich in arginine residues which were apt to be digested by trypsin. The proteolytic peptides of C-terminal of 9G8 were too small to be determined by mass spectrometry. Therefore, it is difficult to detect the acetylation status on lysine residue in the middle and C-terminal region of 9G8.

In conclusion, we have indicated that Sirt1 interacted with 9G8 and deacetylated 9G8 at K24 both in vitro and in live cells. Sirt1 promoted tau exon 10 inclusion, whereas 9G8 inhibited tau exon 10 inclusion. Overexpression of Sirt1 prevented 9G8-promoted 3R-tau expression. Therefore, co-expression of Sirt1 and 9G8 regulated the ratio of 3R-tau/4R-tau nearly to 1. Resveratrol alleviated the dysregulation of tau exon 10 splicing in Htau mice and rescued the defect in spatial memory of Htau mice. Our findings provide novel insights into the molecular mechanism underlying regulation of tau exon 10 splicing and further our experiments aiming at therapeutics for tauopathies.

## MATERIALS AND METHODS

### Animals

Htau mice (STOCK Mapt^tm1 (EGFP)Klt^ Tg (MAPT) 8cPdav/J) (#005491) and tau-KO (mtau^-/-^ littermates) (#004779) were purchased from the Jackson Laboratories (Bar Harbor, ME, USA). Mice were housed in groups of 3 to 6 littermates in individually cages and had free access to water and food. The mice were treated with resveratrol for 7 months at the age of 5 months. Then the mice were subjected to behavioral tests and followed with biochemical analyses. The animal care and experimental protocols were approved by Animal Care and Use Committees of Nantong University.

### Drug

Resveratrol was purchased from Tocris Company (Bristal, UK) and was dissolved in DMSO to prepare a stock solution at 100 nM. The drug was diluted with drinking water and the daily resveratrol consumption was ~3 mg per mouse. The drinking water was refreshed every 3 days.

### Plasmids and antibodies

pCEP4/9G8-HA was from Dr. Tarn of the Institute of Biomedical Sciences, Academia Sinica, Taiwan. pCI/SI9-SI10 contains a tau minigene, SI9/SI10, comprising tau exons 9, 10, and 11, part of intron 9 and intron 10 [35]. Mouse monoclonal anti-SIRT1 and anti-acetylated-lysine antibody were from Cell Signaling Technology (Danves, MA, USA). Rabbit polyclonal anti-HA, mouse monoclonal anti-HA and mouse monoclonal anti-actin antibody were from Sigma (St. Louis, MO, USA). Mouse monoclonal anti-Myc, tetramethyl rhodamine isothiocyanate (TRITC)-conjugated goat anti-rabbit IgG, fluorescein isothiocyanate (FITC)-conjugated goat anti-mouse IgG and human siRNA of SIRT1 were from Santa Cruz Biotechnology (Santa Cruz, CA, USA). Peroxidase-conjugated anti-mouse and anti-rabbit IgG were obtained from Jackson ImmunoResearch Laboratories (West Grove, PA, USA). The ECL kit was from ThermoFisher Scientific (Rockford, IL, USA).

### Plasmid construction and DNA mutagenesis

pcDNA3.1/SIRT1 was constructed by subcloning SIRT1 coding region which was PCR amplificated from Flag-SIRT1 plasmid purchased from Addgene (Cambridge, MA, USA) into mammalian expression vector pcDNA3.1 by *BamH*I and *Not*I. Mutant of 9G8_K24R_ was created by site-directed mutagenesis using KOD-Plus-Mutagenesis kit (TOYOBO, Osaka, Japan) with primers (forward, 5’- gga act ggc gct ggc AGA gga gag tta gaa agg gct ttc agt t-3’, and reverse, 5’- cct ttc taa ctc tcc TCT gcc agc gcc agt tcc cag gtt acc a-3’) and confirmed by DNA sequence analysis.

### Cell culture and transfection

HEK-293T, HEK-293FT and HeLa cells were maintained in Dulbecco’s modified Eagle’s medium supplemented with 10% fetal bovine serum (Invitrogen, CA, USA) at 37 °C (5% CO_2_). Transfections were performed with Lipofectamine 2000 (Invitrogen, CA, USA), Lipofectamine 3000 (Invitrogen, CA, USA) or FuGene 6 (Promega, WI, USA), according to the manufacturer’s instructions.

### Co-immunoprecipitation

HEK-293T cells were co-transfected with pcDNA3.1/SIRT1 and pCEP4/9G8 for 48 h. The cells were washed twice with phosphate-buffered saline (PBS) and lysed by sonication in lysate buffer (50 mM Tris-HCl, pH7.4, 150 mM NaCl, 50 mM NaF, 1 mM Na_3_VO_4_, 2 mM EDTA, 1 mM phenylmethylsulfonyl fluoride, 2 μg/ml aprotinin, 2 μg/ml leupeptin, and 2 μg/ml pepstatin). Insoluble materials were removed by centrifugation. Protein G beads were incubated with mouse anti-HA overnight at 4 °C, and then the antibody bound beads were incubated with the cell lysate. After a 4 h incubation at 4 °C, the beads were washed with lysate buffer twice and with Tris-buffered saline twice, and bound proteins were eluted by boiling in Laemmli sample buffer. The samples were subjected to western blot analysis with the indicated primary antibodies.

### Co-localization study

HeLa cells were plated in 24-well-plates onto coverslips 1 day prior to transfection at 30–40% confluence. The cells were transfected with HA-tagged 9G8 constructs or co-transfected with SIRT1 as described above. Two days after transfection, the cells were washed with PBS and fixed with 4% paraformaldehyde in PB for 30 min at room temperature. After washing with PBS, the cells were blocked with 10% goat serum in 0.2% Triton X-100/PBS for 2 h at 37 °C and incubated with rabbit anti-HA (1:200) and mouse anti-Myc (1:500) overnight at 4 °C. After washing with PBS and incubation with secondary antibodies (TRITC-conjugated goat anti-rabbit IgG and FITC-conjugated goat anti-mouse IgG, 1:200), the cells were washed extensively with PBS and incubated with 5 μg/ml Hoechst 33342 for 5 min at room temperature. The cells were washed with PBS, mounted with Fluoromount-G, and visualized with a Leica TCS-SP5 dual photon laser-scanning confocal microscope.

### Quantitation of tau exon 10 splicing by reverse transcription-PCR (RT-PCR)

Total cellular RNA was isolated from cultured cells by using an RNeasy mini kit (Qiagen, GmbH, Germany). Six hundred ng of total RNA was used for first-strand cDNA synthesis with oligo (dT)_18_ by using an Omniscript reverse transcription kit (Qiagen, GmbH, Germany). PCR was performed by using Prime-STAR^TM^ HS DNA Polymerase (Takara Bio Inc., Otsu, Shiga, Japan) with primers (forward 5’ GGT GTC CAC TCC CAG TTC AA 3’ and reverse 5’ CCC TGG TTT ATG ATG GAT GTT GCC TAA TGA G 3’) to measure alternative splicing of tau exon 10 of mini-tau gene under conditions: denaturation for 5 min at 98 °C was followed by 30 cycles with denaturation for 10 sec at 98 °C, annealing for 15 sec at 55 °C, polymerization for 30 sec at 72 °C and a final extension for 10 min at 72 °C. The PCR products were resolved on 1.5% agarose gels and quantitated using the Molecular Imager system (Bio-Rad, CA, USA).

### Deacetylation assay in vitro

HEK-293T cells were transfected with pCEP4/9G8-HA. After 48 h, cell lysate was incubated with mouse anti-HA antibody conjugated protein G beads. The immunoprecipitated complexes was washed with lysis buffer three times and twice with deacetylation buffer (50 mM Tris-HCl, pH 9.0, 0.5% glycerol, 50 mM NaCl, 4 mM MgCl_2_, 0.5 mM DTT, 0.1 mM PMSF, 0.02% NP-40). The immune complexes were subsequently incubated with Sirt1 (Sigma, MO, USA) and 50 μM NAD^+^, or only with NAD^+^ in 30 μl deacetylation buffer for 2 h at 37 °C. The reaction was terminated with SDS sample buffer and subjected to SDS-PAGE. Acetylation level of 9G8 was examined by immunoblotting developed with anti-acetylated-lysine antibody and anti-HA antibody.

### Mass spectrometry

pCEP4/9G8-HA was co-transfected with pcDNA3.1/SIRT1 or pcDNA3.1 into HEK-293T cells. After 72 h transfection, cell lysate was incubated with Pierce™ anti-HA Magnetic Beads (Thermo Scientific^TM^). The immunoprecipitated products were separated in SDS-PAGE and stained by silver staining. The HA-9G8–containing gel piece was in-gel tryptic–digested. Proteolytic peptides were extracted from the gel, followed by TiO2 IMAC enrichment for the acetylpeptides. The resulting fraction was concentrated and reconstituted in 10 μl of 5% formic acid for LC-MS/ MS analysis.

### Elevated plus-maze

12 months old Htau and tau-KO mice were subjected to general behavioral test. The elevated plus-maze consisted of four arms (30×5 cm) connected by a common 5×5 cm center area. Two opposite facing arms were open (open arms, OA), whereas the other two facing arms were enclosed by 20 cm height walls (closed arms, CA). The entire plus-maze was elevated on a pedestal to a height of about 80 cm above floor level. During a single 8-min session, an animal was placed onto the central area. Any-maze (Stoelting Co., IL) video tracking system detected the presence of the animal and the time it spent in the different zones of maze-arms. For each animal, the number of CA entries, OA entries, and amount of time spent in CA and OA were recorded. The percentage of time spent in OA and the entries into OA were recorded and calculated to evaluate anxiety-like behavior of animals.

### Open-field activity test

After elevated plus-maze test, the mice were tested by open-field activity. The testing classic open field was a square arena, 50×50 cm, with 40 cm high walls. The mice were individually subjected to the test for 15 min. The arena was divided into 9 equal virtual squares. The general exploration and locomotor activity were recorded. Amount of time spent in the center of the arena and the entries to center arena were recorded as an additional measure of anxiety.

### Accelerating Rota-rod test

The mice were subjected to accelerating Rota-rod-test by giving each mouse 2 sessions of 3 trials each with the motor in accelerating mode (Ugo BasileSrl, Italy). The rotating speed increased steadily, at a rate of 0.02 cm/s, from 4 to 40 rpm. The latency to fall off the Rota-rod was calculated. Inter-trial intervals were 10-15 min for each mouse.

### Spatial learning and memory task in the water-maze

After general behavioral tests, spatial learning and memory test was conducted by Morris water maze (MWM). The test was performed in a 180 cm-diameter, 60 cm-height circular tank filled with water (23 °C ± 2) made opaque by adding white non-toxic paint. The maze was divided into four equal quadrants by two principal axes with each one bisecting the maze perpendicular to the other one. A 13 cm-diameter platform submersed 1 cm under the water surface was placed in the center of one of the four imaginary quadrants of the tank and maintained in the same position during all trials. 90 s was given for each mouse to find the platform. If the mouse did not find the platform in 90 s, it was gently guided to it. At the end of each trial, the mouse was left on the platform for 20 s. Three such acquisition trials were given on each day for 4 consecutive days. Each mouse was subjected to 12 trials totally corresponding to a partial training of the spatial reference memory task. A test for retention (probe trial) was given 24 h after the last day of training. For probe trial, the mouse was allowed to swim in the tank without the escape platform. The time and the distance swum to reach the escape platform were measured through an automated tracking system (Smart video tracking system, Panlab; Havard Apparatus).

### Statistical analysis

Where appropriate, the data are presented as means ± S.D. Data points were compared by the unpaired two-tailed Student’s *t*-test for two groups’ comparison, one-way ANOVA and two-way ANOVA. The calculated *p*-values are indicated in the figures.
